# The rise in the number of long-term survivors from different diseases can slow the increase in life expectancy of the total population

**DOI:** 10.1186/s12889-020-09631-3

**Published:** 2020-10-07

**Authors:** Marcus Ebeling, Anna C. Meyer, Karin Modig

**Affiliations:** 1 Max Planck Institute for Demographic Research, Konrad-Zuse-Straße 1, 18055 Rostock, Germany; 2 University of Rostock, Rostock, Germany; 3 Unit of Epidemiology, Institute of Environmental Medicine, Karolinska Institutet, SE-17177 Stockholm, Sweden

**Keywords:** Disease prevalence, Life expectancy, Disease prevention, Failure of success, Decomposition method, Survival after diagnosis

## Abstract

**Background:**

Recent improvements in life expectancy in many countries stem from reduced mortality from cardiovascular disease and cancer above the age of 60. This is the combined result of decreased incidence and improved survival among those with disease. The latter has led to a higher proportion in the population of people with a past history of disease. This is a group with higher mortality than the general population. How growing shares of persons with past history of disease and improved survival with disease have affected changes in life expectancy of the total population is the objective of this paper.

**Methods:**

Using register data for the total Swedish population, we stratified the population based on whether individuals have been diagnosed with myocardial infarction, stroke, hip fracture, colon cancer, or breast cancer. Using a novel decomposition approach, we decomposed the changes in life expectancy at age 60 between 1994 and 2016 into contributions from improved survival with disease and from changes in proportion of people with past history of disease.

**Results:**

Improvements in survival from disease resulted in gains of life expectancy for the total population. However, while the contributions to life expectancy improvements from myocardial infarction, stroke and breast cancer were substantial, the contributions from the other diseases were minor. These gains were counteracted, to various degrees, by the increasing proportion of people with raised mortality due to a past history of disease. For instance, the impact on life expectancy by improved survival from breast cancer was almost halved by the increasing share of females with a past history of breast cancer.

**Conclusion:**

Rising numbers of survivors of different diseases can slow the increase in life expectancy. This dynamic may represent the costs associated with successful treatment of diseases, and thus, a potential “failure of success.” This dynamic should be considered when assessing mortality and life expectancy trends. As populations are aging and disease survival continues to improve, this issue is likely to become even more important in the future.

## Background

Recent gains in life expectancy have been primarily driven by mortality improvements at older ages [1]. Declining disease incidence and improved survival with diseases have been instrumental for this development. Tremendous improvements in the incidence and the survival from cardiovascular disease and different types of cancer, as well as improvements in the life expectancy of diabetics, are only a few of the achievements of a range of successful interventions in this context [2–5]. There are many more examples of advancements in primary and secondary prevention of various diseases [6–10].

Survival after diagnosis has generally improved at a faster pace than disease incidence has come down. This has led to growing proportions of persons with a past disease history, or in other words, the prevalence of many diseases has grown [11]. This development has altered the composition of the total population, as the proportion of individuals with a disease history has risen. Although the prognosis for patients of various diseases has improved and led to higher survival chances, these individuals may face a higher mortality risk later on, at higher ages, than their disease-free counterparts. This may potentially slow down the overall mortality improvement, or life expectancy increase in the population.

It is possible that this mechanism has already contributed to the recently observed slowdown in life expectancy increases in many low-mortality countries [12]. Even if this is not the case, ongoing population aging will likely continue to generate increasing proportions of individuals with a disease history in the future, thereby making the interplay of mortality after diagnosis and the prevalence proportions even more relevant for life expectancy changes at the national level. It is also generally unknown, which role improved mortality after diagnosis plays as driver of life expectancy change. Given these trends, and the important role that life expectancy (or life years gained) plays in public health and health policy decision-making, it is crucial that we understand how these dynamics are affecting changes in overall life expectancy levels [13, 14]. The dynamics also link to a debate about the potential downsides of medical and technological advancements [15]. This article aims to contribute to this topic by investigating *how improved survival from major diseases has contributed to gains in general life expectancy in Sweden, and whether these gains have been offset by increases in the prevalence of the respective diseases.*

## Methods

### Data

This study is based on data drawn from several Swedish population registers, namely the register of the total population, the National Patient Register and the Swedish Cancer Register [16–20]. The data covers all men and women over the age of 60 who were residing in Sweden at any point in time during 1994 and 2016. The total population was divided into subpopulations, consisting of individuals with a history of either myocardial infarction, stroke, hip fracture, colon cancer, or breast cancer (only females). The five diseases were selected because they together constitute a majority of the disease panorama in Sweden as well as in other low-mortality countries. Furthermore, they have faced changing incidence as well as survival rates the past decades. They can also readily be studied with register data because they have a clear onset, result in hospital admission, and are usually accurately diagnosed [16, 19, 20]. Moreover, these diseases do not only represent different pathogeneses, they also have different risk factors and prognoses. Thus, they provide a meaningful basis to investigate the major possibilities of how mortality after diagnosis and disease prevalence can affect life expectancy. We have chosen to examine first events only. Each disease was selected separately, meaning that individuals could belong to more than one disease group. The data are available up to the end of calendar year 2016.

To capture how mortality improvements during the more acute phase of the disease versus the long-term mortality improvements contributed to the change in overall life expectancy, we stratified the subpopulation of each disease into two subgroups, which we labeled recent and distant cases. For myocardial infarction, hip fracture, and stroke, all individuals who were diagnosed in the 3 years prior to the calendar year were considered recent cases. For breast and colon cancer, all individuals who were diagnosed in the 5 years prior to the calendar year were classified as recent cases. These time frames have been chosen somewhat arbitrarily, though it is worth noting that for many types of cancer, individuals are considered cured after spending 5 years in remission. All individuals who were diagnosed at earlier points in time are considered distant cases. Altogether, we thus stratified the total population for each disease separately into three subpopulations: recent cases, distant cases, and the disease-free population. The disease-free population contains the individuals that have not been diagnosed with the respective analyzed disease.

We calculated the age-specific proportion, or prevalence, by dividing the subpopulation-specific person-years lived by the total number of person-years lived. To reduce random fluctuations, we smoothed the death rates over age and time with a widely-used two-dimensional smoothing procedure [21].

### Decomposing life expectancy differences

The age-specific death rates observed for the total population can be understood as weighted averages of the age-specific death rates across the subpopulations [22]. The mortality of these subpopulations is linked to the mortality of the total population by two factors: namely, the subpopulation-specific mortality (I) and the share of the subpopulation in the total population (II) [23]. The mortality level of a subpopulation contributes to a higher or lower overall mortality depending on whether the level is above or below the average mortality level. This effect on the mortality of the total population is mediated by the size of the subpopulation; or, more concretely, by the proportion of the subpopulation on the total population. Accordingly, the effect on the mortality of the total population rises as the proportion of the subpopulation increases.

By stratifying the total population by individual disease histories, subpopulation-specific mortality can be interpreted as mortality after diagnosis, whereas the proportion of the subpopulation can be understood as the age-specific prevalence proportion. This approach allows us to decompose the change in life expectancy into the contributions of changing mortality after diagnosis and changing prevalence proportions. We perform this decomposition for each disease separately.

In a first analysis, we assign deaths to the respective groups of recent or distant cases and to disease-free individuals. This procedure is similar to cause-of-death distinction, with the difference that here disease diagnosis is the distinction criteria. In a second analysis, we disaggregate the contributions of the groups in the previous setting and calculate the impact of changes in mortality after diagnosis and changes in the proportion of persons with a past disease history. To estimate the contributions of each component, we employ a general decomposition algorithm (see Additional file 1) [24].

Life expectancy is measured by annual average life years lived between ages 60 and 104, the last available age. This measure is also called partial life expectancy (see Additional file 1). The magnitude of this measure is very similar to the actual remaining life expectancy at age 60, since it only misses the contributions of the population above age 105, which is relatively small. In the remainder of the article, we will refer to this measure simply as remaining life expectancy.

## Results

### Change in the population composition

Table 1 shows the distribution of person-years lived and deaths across the two different subgroups (recent and distant cases) as well as for the disease-free over the study period. The distribution of person-years lived clearly shows that the amount of person-years lived by people with a past disease history has increased between 1994 and 2016. Accordingly, for all diseases and both sexes, the share of person-years lived by the disease-free population has decreased over time. At the same time, the proportion of person-years lived by long-term survivors (distant cases) increased between both years, while the proportion of recent cases declined for all diseases, except for colon cancer and breast cancer.

**Table 1:** Proportions on all person-years lived and all deaths in percent for major diseases by recent cases, distant cases and the disease-free subpopulation at ages 60 to 104 combined, years 1994 and 2016, Sweden, males and females

		Person-years lived						Deaths					
		1994			2016			1994			2016		
Disease	Sex	Recent	Distant	Dis.-free	Recent	Distant	Dis.-free	Recent	Distant	Dis.-free	Recent	Distant	Dis.-free
Myo. infarction	F	1.18	2.32	96.5	0.88	3.09	96.03	15.07	6.44	78.49	6.7	9.25	84.05
	M	2.31	6.19	91.5	1.61	7.73	90.67	19.14	11.95	68.91	8.26	16.09	75.65
Stroke	F	1.74	1.84	96.42	1.27	3.52	95.21	14.89	7.15	77.96	9	12	79
	M	2.25	2.55	95.2	1.59	4.9	93.51	12.31	7.75	79.94	7.96	13.67	78.36
Hip fracture	F	2.22	1.97	95.81	1.65	3.04	95.31	10.06	8.03	81.9	9.39	14.67	75.94
	M	0.9	0.67	98.43	0.79	1.39	97.82	5.47	2.47	92.06	6.38	5.72	87.9
Colon cancer	F	0.39	0.56	99.05	0.61	0.96	98.43	2.06	1.01	96.93	2.24	2.2	95.56
	M	0.44	0.48	99.08	0.69	0.89	98.42	2.02	1.04	96.93	2.54	2.12	95.34
Breast cancer	F	1.33	2.58	96.09	1.78	5.34	92.88	2.65	4.47	92.88	2.35	7.8	89.85

Notes: The recent cases are those with a diagnosis in the previous 3 years (myocardial infarction, stroke, hip fractures) or in the previous 5 years (colon and breast cancer). The distant cases are those in the respective residual group. The disease-free population refers to the share of the population who had not been diagnosed with the respective disease. Data: Swedish National Patient Register and Swedish National Cancer Register. Own calculations

Over time the proportions of deaths from the disease-free subpopulations have declined, with the exception of myocardial infarction and stroke among females. The strong decline in the proportion of deaths from recent myocardial infarction-cases is the main driver for the increase of the proportion of deaths from the disease-free subpopulation. This is seen from the increase in distant cases of myocardial infarction over time. The proportion of deaths actually increased from distant cases for all diseases, while the opposite is true for recent cases with the exception of colon cancer and hip fracture among males.

### Decomposition of life expectancy change into the contributions of deaths by recent and distant cases

**Fig. 1:**
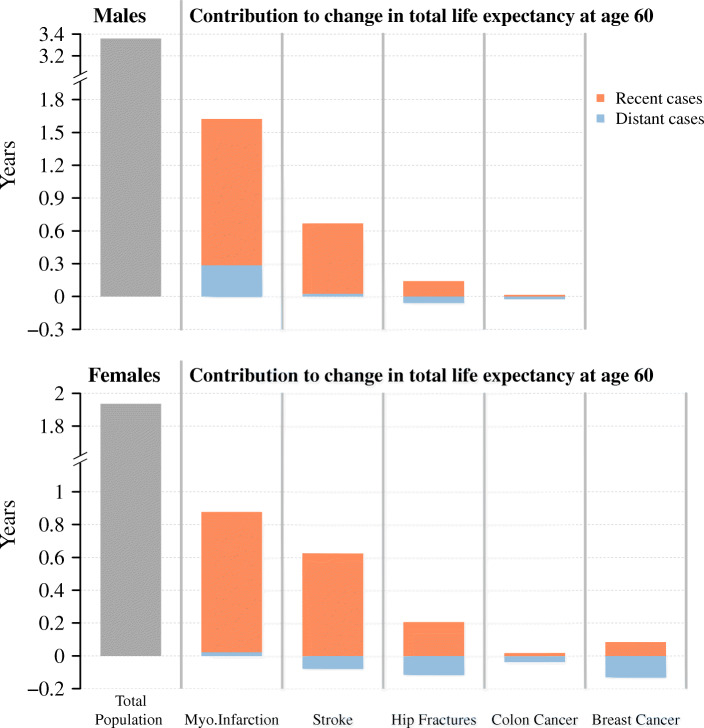
Contributions of recent and distant cases of different diseases to the increase in life expectancy at age 60 between 1994 and 2016, Sweden, males and females. Notes: Life expectancy at age 60 is defined as the average person-years lived between ages 60 and 104. The respective contributions are the sum across the respective age-specific contributions. The recent cases are those with a diagnosis in the previous three years (myocardial infarction, stroke, hip fracture) or in the previous five years (colon and breast cancer). The distant cases are those in the respective residual group. The disease-free population refers to the share of the population who had not been diagnosed with the respective disease. Results for the disease-free population can be found in Additional file 1. Data: Swedish National Patient Register and Swedish National Cancer Register. Own calculations

Figure 1 shows how each disease-specific subgroup contributed to the life expectancy increase between 1994 and 2016, separately for males and females. The total change in life expectancy during the observation period is depicted by the gray bar on the left. The contributions of changes in deaths by recent and distant cases are depicted by the respective orange and blue bar. The contribution of the respective disease-free group can be seen in Additional file 1.

Remaining life expectancy at age 60 increased by around 3.36 years for males and by around 1.93 years for females during the observation period. The disease-specific decompositions revealed that mortality improvements after myocardial infarction and stroke have been instrumental for the increase of life expectancy, especially mortality improvements occurring within 3 years from the diagnosis. The contributions from mortality improvement of more distant cases have been moderately negative to virtually zero, with the exception of myocardial infarction, for which the contributions are found to be 0.3 years for males, and marginally positive for females.

**Fig. 2:**
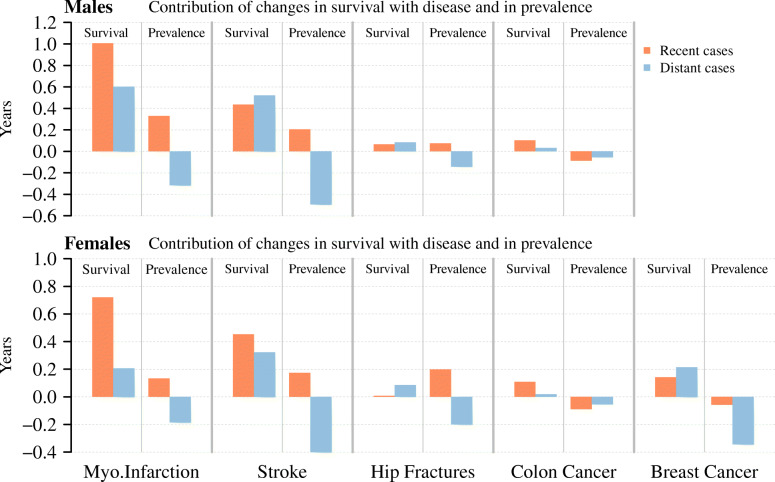
Contributions of recent and distant cases of different diseases to the increase in life expectancy at age 60 between 1994 and 2016 by survival with disease and by prevalence, Sweden, males and females. Notes**:** Life expectancy at age 60 is defined as the average person-years lived between ages 60 and 104. The respective contributions are the sum across the respective age-specific contributions. The recent cases are those with a diagnosis in the previous 3 years (myocardial infarction, stroke, hip fracture) or in the previous 5 years (colon and breast cancer). The distant cases are those in the respective residual group. The disease-free population refers to the share of the population who had not been diagnosed with the respective disease. Results for the disease-free population can be found in Additional file 1. Data: Swedish National Patient Register and Swedish National Cancer Register. Own calculations

The contributions shown in Fig. 1 are the combined result of improvements in survival with the disease and changes in the proportion of the respective group on the total population. To understand how each of the two factors affected these contributions, it is necessary to further disaggregate the disease- and subgroup-specific estimates. The respective results are shown in Fig. 2. The figure shows the contributions of each disease after separating survival improvement and changes in prevalence for recent and distant cases, respectively.

For both recent and distant cases and for all diseases, improved survival contributed to an increase in life expectancy. The disaggregation again underpins the central importance of improved survival in the acute phase of myocardial infarction and stroke for the overall life expectancy improvement observed in Sweden between 1994 and 2016. For breast cancer, the contributions from improved survival are larger from distant cases than from recent cases. Hip fracture and colon cancer contributed the least to the improvement in life expectancy.

Figure 2 also emphasizes the strong counteracting effect from the increase in the prevalence of distant cases. Throughout all analyzed diseases and both sexes, the increasing prevalence of distant cases revealed negative contributions, and thus, would have resulted in life expectancy declines, despite all other contributions. The strong effect is, for instance, visible for stroke among males, where improved survival is almost completely offset by the increase in prevalence of distant cases. This result has been hidden in the results presented in Fig. 1 because of the impact of changes in the prevalence of long-term survivors. It is important to note that increasing prevalence makes negative contributions to the overall changes in life expectancy and vice versa.

Moreover, for the recent cases of hip fracture, we can observe that the positive contribution of this subgroup was entirely driven by declines in the prevalence proportion (see Additional file 1), while mortality after diagnosis remained largely unchanged between 1994 and 2016. Recent cases of myocardial infarction and stroke are more complex examples, as their prevalence proportions declined below age 90 but increased at ages 90+. However, the positive age-specific contributions below age 90 were higher than the negative age-specific contributions above age 90, which turned the overall contribution of changes in prevalence of recent cases positive.

### Summary of contributions to life expectancy increase

So far, we have addressed the effect of changing prevalence to the disease-specific contribution to life expectancy change. But because the subgroup of recent and distant cases does not change independently of the disease-free subpopulation, understanding the impact of the change in the total composition on life expectancy thus requires an interpretation of the sum, which includes the three. If the sum is positive, it means that changes in the composition contributed to life expectancy increases by reducing the number of individuals with disease history. A negative value suggests that changes in the composition prevented even greater increases in life expectancy, through opposite dynamics.

Table 2 summarizes the total impact of all components on the change in remaining life expectancy. For myocardial infarction among males, improved survival for both recent and distant cases contributed 1.61 years to the total increase in life expectancy. This corresponds to 48.5% of the total increase in life expectancy. For females, the corresponding figures are 0.93 years and 48%. In both cases, this is almost half of the total change in life expectancy between 1994 and 2016. The remaining contribution to the increase in life expectancy stems from improved survival among those free of myocardial infarction. The changing population composition had a small positive contribution of 0.15 years for males, and a neglectable effect for females, 0.03 years, to the total life expectancy change. By contrast, for stroke, breast, or colon cancer, we observe that the compositional changes prevented stronger life expectancy increases, and thus, potentially slowed overall increases in the average remaining life years at age 60. For instance, the shift towards a higher proportion of females that have a past history of breast cancer offsets almost half of the contribution from survival improvements with this disease.

**Table 2:** Contributions of five major diseases to the increase in life expectancy at age 60 between 1994 and 2016 by improved survival of the subpopulation with disease history and the disease-free subpopulation and by changing population composition in years, Sweden, males and females

		Changing survival		
Disease	Sex	After diagnosis	Disease-free pop.	Changing population composition
Myo. infarction	F	0.93 [48.05%]	0.97 [50.51%]	0.03 [1.44%]
	M	1.61 [48.5%]	1.56 [46.93%]	0.15 [4.58%]
Stroke	F	0.78 [39.94%]	1.27 [65.42%]	−0.1 [−5.36%]
	M	0.96 [28.42%]	2.54 [75.47%]	−0.13 [−3.89%]
Hip fracture	F	0.09 [4.76%]	1.82 [93.56%]	0.03 [1.68%]
	M	0.15 [4.43%]	3.26 [96.33%]	−0.03 [−0.76%]
Colon cancer	F	0.13 [6.55%]	1.88 [97.41%]	−0.08 [−3.96%]
	M	0.13 [3.96%]	3.32 [98.29%]	−0.08 [−2.25%]
Brest cancer	F	0.36 [18.43%]	1.72 [89.32%]	−0.15 [−7.75%]

Notes: Life expectancy at age 60 is defined as the average person-years lived between ages 60 and 104. The respective contributions are the sum across the respective age-specific contributions and the related subpopulations. Summing the number in each line leads to the respective total change in life expectancy between 1994 and 2016, or obviously, 100%. The number for the changing population composition includes both the subpopulation with a disease history and the disease-free population. Data: Swedish National Patient Register and Swedish National Cancer Register. Own calculations

## Discussion

In this article, we presented to which extent mortality improvement for five major diseases has contributed to the increase in life expectancy in the past two decades in Sweden. Furthermore, we quantified to what extent these gains have been offset by increases in the prevalence of respective diseases. We found that the net effect of survival improvement and increasing prevalence has resulted in a positive contribution to life expectancy development for myocardial infarction, stroke, and (albeit to a much smaller extent) hip fracture. However, for breast and colon cancer, the net effect was not only zero, it was even slightly negative. This finding may appear to contradict to existing evidence of improvements in cancer survival [4]. However, it does not. The improvement in cancer survival at the ages when the cancer occurs up to 5 years after diagnosis has indeed contributed positively to life expectancy. The long-term survivors, however, carry with them a higher mortality rate than the total population when they reach higher ages. This could decelerate the decline in the average mortality rate, and in some cases even the increase in total life expectancy. Does this mean that medical advancements have resulted in improvements in short-term survival only? No. Survival after diagnosis improved for all diseases for both recent and distant cases, although the contributions to life expectancy from improved short-term survival were still much higher. However, even if all groups improved their survival and life expectancy, given the negative impact from the increase in long-term survivors of three out of five major diseases for females and for three out of four major diseases for males, it could be argued that this slowed the increase in life expectancy in the general Swedish population.

Studies usually aim to assess the epidemiology of diseases using measures such as disease-specific survival and prevalence. In contrast, our study aims to link the epidemiology of the disease to the change in overall population health, in this case, to life expectancy of the total population. By only estimating mortality among disease-specific populations, we can only speculate about the impact on the total population. In order to make a clear quantitative link, the share of the disease-specific population must be considered. This is because this share translates disease-specific measures to the level of the total population. For our study, this means that the increasing proportion of individuals with disease history not only makes up a larger share of the total population, it also means that the share of deaths coming from this subpopulation increased. The only force that can prevent this development is improved survival within this population, which would lower the number of deaths coming from this subpopulation. In our study, we see this for some diseases and ages, but not all. That is why the total effect is positive for some of the diseases and negative for others.

Our analysis is the first to demonstrate the composition and the mortality dynamics that occur within the total population when effective disease prevention efforts are undertaken. Most previous research used causes of death or changes in age-specific mortality to capture the effects of health dynamics on longevity change [25–27]. Among the drawbacks of relying on causes of death are that the quality of the data is limited and the diseases are identified retrospectively [28, 29]. We therefore attempted to capture these dynamics using disease diagnosis, which reflects the disease progression over an individual’s lifetime. However, as a diagnosis also does not capture the entire spectrum of the health consequences of a disease, our ability to draw conclusions regarding the actual health burden originating from a specific diagnosis is limited.

In addition, while a disease diagnosis marks the threshold between being healthy and being ill, it cannot capture the progress that has been made by avoiding or preventing the disease. Moreover, for the sake of feasibility and interpretability, we decided to not include in our analysis comorbidity, which is an important factor, particularly when examining elderly populations. By excluding comorbidity, we also did not account for competing risks that play a role in developing a certain disease and also in dying from a certain disease. These are certainly subjects for future research.

Although a consequence of successful medical interventions, the increasing share of individuals with a disease history could pose a challenge to further increases in average longevity. However, it needs to be stressed that this “cost” is a byproduct of saving lives, and hence should be seen as inherently positive. The question of whether this development is considered a cost or a “failure of success” is only raised in the context of life expectancy [15]. Nevertheless, reducing the number of individuals who develop diseases is becoming increasingly important in order to maintain a continuing rise in life expectancy.

The paradoxical relationship between changes in disease prevalence and longevity improvements may also lead us to reflect critically on the use of life expectancy as a measure for evaluating health policies. This and similar measures tend to overlook the progress that has been made by various subpopulations with a disease history. Alternative measures might rely on approaches that are less sensitive to population composition, or, if this is not possible, should at least acknowledge potential opposing dynamics. Although these dynamics were assessed for Sweden only, they are generally applicable to populations with disease spectrums that are dominated by non-communicable and chronic diseases.

## Conclusion

Improved survival from major diseases, especially myocardial infarction, has been a driver of life expectancy increases at the national level the past decades. However, as survival improves, prevalence rises and excess mortality in subpopulations with a disease history gains a greater impact on mortality in the total population. Although the effect of this has been small, it still had a decelerating effect on the life expectancy increase. As populations are aging and disease survival continues to improve, this issue is likely to become more important in the future. Ignoring such compositional changes can lead us to overlook the progress that has been made.

## Supplementary information

**Additional file 1.** Supplemental materials: The rise in the number of long-term survivors from different diseases can slow the increase in life expectancy of the total population. Supplemental materials and further results for the presented study.

## Data Availability

This study is based on national registers in Sweden and the datasets contain sensitive information. Access to full data is available upon request from Karin Modig, given that the person that is interested to use it receives ethical vetting and approval from the data owners, the National Board of Health and Welfare in Sweden and Statistics Sweden. However, some aggregated tables can be provided by the corresponding author upon request. The code for estimation is available upon request from the corresponding author.
