# Supplementary material for: The rise in the number of long-term survivors from different diseases can slow the increase in life expectancy of the total population
Source: BMC Public Health. 2020 Oct 7;20:1523. doi: 10.1186/s12889-020-09631-3 (PMC7542716; doi:10.1186/s12889-020-09631-3)
Supplement: Supplementary file 1 — Additional file 1. Supplemental materials: The rise in the number of long-term survivors from different diseases can slow the increase in life expectancy of the total population. Supplemental materials and further results for the presented study. [file 12889_2020_9631_MOESM1_ESM.pdf]

Supplemental Materials:
The rise in the number of long-term survivors from different diseases can slow the increase in life expectancy of the total population

Marcus Ebeling* Anna Meyer† Karin Modig†

ICD codes used in the analysis

Table 1: **Concordance table for coding of primary diagnosis.** *Information on stroke, myocardial infarction and hip fracture is retrieved from the National Patient Register. Information on colon and breast cancer is based on the Swedish Cancer register. For cancer, ICD-7 codes are used because translating them to ICD-10 codes is less complicated.*

Disease	Codes
Stroke	431, 4334, 436 (ICD-9), I61, I63, I64 (ICD-10)
Myocardial infarction	410 (ICD-9), I21-22 (ICD-10)
Hip fracture	820 (ICD-9), S72 w/o S72.3, S72.4, S72.9 (ICD-10)
Colon cancer	153 (ICD-7), C18 (ICD-10)
breast cancer	170 (ICD-7), C50 (ICD-10)

Construction of partial life expectancy

The calculation of partial life expectancy follows the general calculation of life expectancy. Accordingly, partial life expectancy also requires the calculation of age-specific survival fractions, l_x, and the respective person-years lived within each age interval, L_x. Detailed explanations of how these measures are calculated have been provided elsewhere.[1] In contrast to classic remaining life expectancy, partial life expectancy refers to a specific age range. Thus, the value expresses the average number of life years an individual can expect to live within the respective age range. Accordingly, the partial life expectancy between ages x_1 and x_2, $e^P_{x_1,x_2}$, can calculated by

$$e^P_{x_1,x_2} = \frac{\sum_{x_1=i}^{x_2} L_i}{l_{x_1}}, \text{ where } x_2 > x_1.$$

*University of Rostock, Rostock, Germany and Max Planck Institute for Demographic Research, Rostock, Germany
†Karolinska Institutet, Stockholm, Sweden

Splitting age-specific death rates to decompose life expectancy

The results of this study rest on a decomposition of life expectancy that is based on a split of the age-specific death rate in the death rates of different subpopulations. More concrete, we split each age-specific death rate into a subpopulation who experienced the disease recently (*recent cases*) (up to three or five (cancer types) years prior to the calendar year), a subpopulation whose diagnosis was further in the past (*distant cases*) (more than three or five years prior to the calendar year), and a *disease-free* subpopulation. By interpreting the age-specific death rate,m_x, as a weighted average across subpopulations, this splitting can mathematically be expressed by

$$m_x = \pi_x^{\text{recent cases}} m_x^{\text{recent cases}} + \pi_x^{\text{distant cases}} m_x^{\text{distant cases}} + \pi_x^{\text{disease-free}} m_x^{\text{disease-free}},$$

with π_x being the proportion of the respective subpopulation on the total population at age x, $m_x^{\text{subpop.}}$ being the death rate of the respective subpopulation, and where

$$\pi_x^{\text{recent cases}} + \pi_x^{\text{distant cases}} + \pi_x^{\text{disease-free}} = 1.$$

The death rates of the respective subpopulations with a disease history can be interpreted as mortality after diagnosis. Note that a prevalence proportion is defined as the proportion of the population who were affected by a specific health condition, and, thus $\pi^{\text{recent cases}}$ as well as $\pi^{\text{distant cases}}$ can be respectively interpreted as the prevalence proportion of individuals that were recently diagnosed and individuals who were diagnosed on the more distant. The splitting could be extended to any number of subpopulations.

Based on this splitting and our interpretations, the applied decomposition algorithm[2] decomposes the change in partial life expectancy, Δe^P, as follows

$$\Delta e^P = \underbrace{\Delta\pi + \Delta m}_{\text{recent cases}} + \underbrace{\Delta\pi + \Delta m}_{\text{distant cases}} + \underbrace{\Delta\pi + \Delta m}_{\text{disease-free}},$$

with Δm being the contribution of changing mortality in the respective subpopulation and $\Delta\pi$ being the contribution of changes in the prevalence proportion or the proportion of the respective subpopulation. The different contributions can further be summed to obtain more general contributions:

$$\Delta e^P = \Delta^{\text{recent cases}} + \Delta^{\text{distant cases}} + \Delta^{\text{disease-free}} = \Delta^{\text{with disease}} + \Delta^{\text{disease-free}}.$$

This methodological procedure is certainly not without limitations. The prevalence component in particular has some pitfalls. First, our definition of prevalence is based on person-years lived, and thus deviates from the classical definition of cases divided by the number of persons. By using our definition, we also violate the assumption that prevalence proportion is a stock size that is related to a specific point in time. The issue of how individuals are handled within the year of diagnosis also arises. Within this year, these individuals contribute person-years to both the disease-free and the diagnosed population. This is clearly a problem when relying on the concept of prevalence, as small deviations from a classical prevalence proportion are possible. However, using the prevalence proportion as weights is not necessarily problematic because the contribution to the weights is properly assigned. Moreover, the prevalence proportion as such is not completely free of mortality effects.

For instance, as an increasing share of distant cases would not be possible without considerable increases in survival for diagnoses in the past, some trends are self-evident. In our setting, however, we compare life tables that are each assumed to represent an independent closed population. Thus, the prevalence components are interpretable from a comparative statics perspective .

Detailed overview of decomposition results

Table 2: **Decomposition of the difference in average lived person-years between ages 60 and 104 and the years 1994 and 2016, males and females.** For hip fracture, stroke, and myocardial infarction, the recent cases are defined as those that were diagnosed in the three years prior to the calendar year. For the different types of cancer, the recent cases are defined as those that were diagnosed in the five years prior to the calendar year. Accordingly, the distant cases are those in the respective residual group. Each number is the sum across of the age-specific contributions. Data: Swedish National Patient Register and Swedish National Cancer Register. Own calculations.

	recent cases		distant cases		disease-free	
	Δm_x	$\Delta \pi_x$	Δm_x	$\Delta \pi_x$	Δm_x	$\Delta \pi_x$
**Hip Fractures**			Females			
in years	0.007	0.199	0.085	-0.199	1.823	0.034
% of total change	0.378	10.190	4.380	-10.234	93.563	1.723
			Males			
in years	0.065	0.076	0.084	-0.142	3.255	0.041
% of total change	1.931	2.236	2.499	-4.215	96.332	1.214
**Myocardial infarction**			Females			
in years	0.721	0.133	0.207	-0.184	0.975	0.079
% of total change	37.352	6.902	10.701	-9.558	50.505	4.097
			Males			
in years	1.006	0.329	0.603	-0.315	1.557	0.138
% of total change	30.332	9.911	18.164	-9.503	46.925	4.170
**Stroke**			Females			
in years	0.453	0.173	0.323	-0.400	1.270	0.123
% of total change	23.323	8.927	16.614	-20.624	65.422	6.337
			Males			
in years	0.436	0.205	0.521	-0.494	2.540	0.159
% of total change	12.942	6.080	15.474	-14.679	75.468	4.712
**Colon cancer**			Females			
in years	0.108	-0.090	0.019	-0.055	1.884	0.069
% of total change	5.589	-4.637	0.958	-2.869	97.411	3.547
			Males			
in years	0.102	-0.088	0.031	-0.055	3.316	0.067
% of total change	3.037	-2.597	0.920	-1.644	98.286	1.995
**Breast cancer**			Females			
in years	0.141	-0.058	0.214	-0.344	1.721	0.253
% of total change	7.333	-3.018	11.100	-17.856	89.318	13.122

Decomposition results for varying starting ages

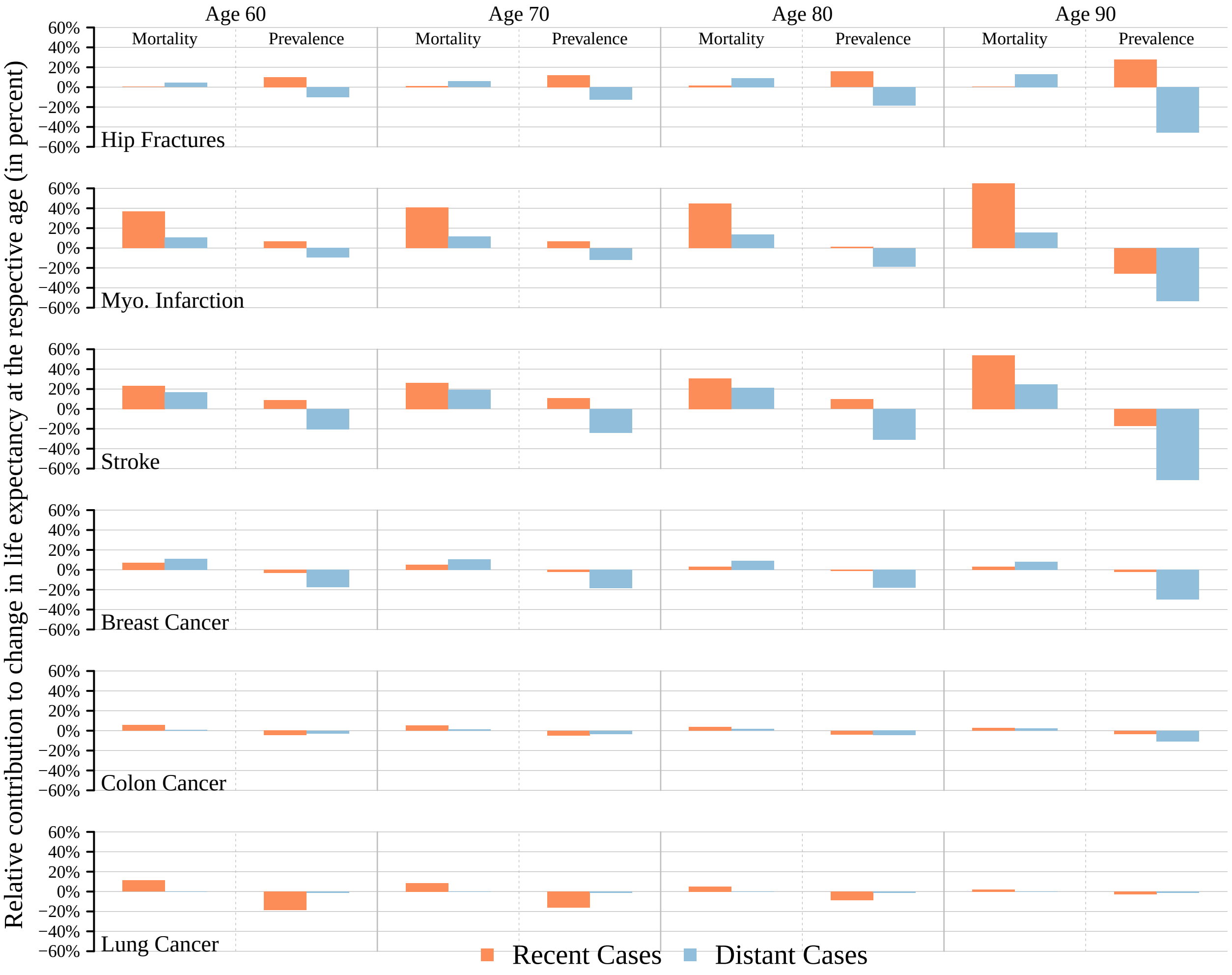

Figure 1: **Decomposition for the difference in remaining life expectancy at ages 60, 70, 80 and 90 between 1994 and 2016 for different diseases, females, Sweden.** The different decompositions rely on different starting ages, and decompose the respective average person-years between the starting age and age 104. For hip fracture, stroke, and myocardial infarction, the recent cases are defined as those that were diagnosed in the three years prior to the calendar year. For the different types of cancer, the recent cases are defined as those that were diagnosed in the five years prior to the calendar year. Accordingly, the distant cases are those in the respective residual group. Each number is the sum across of the age-specific contributions. Data: Swedish National Patient Register and Swedish National Cancer Register. Own calculations.

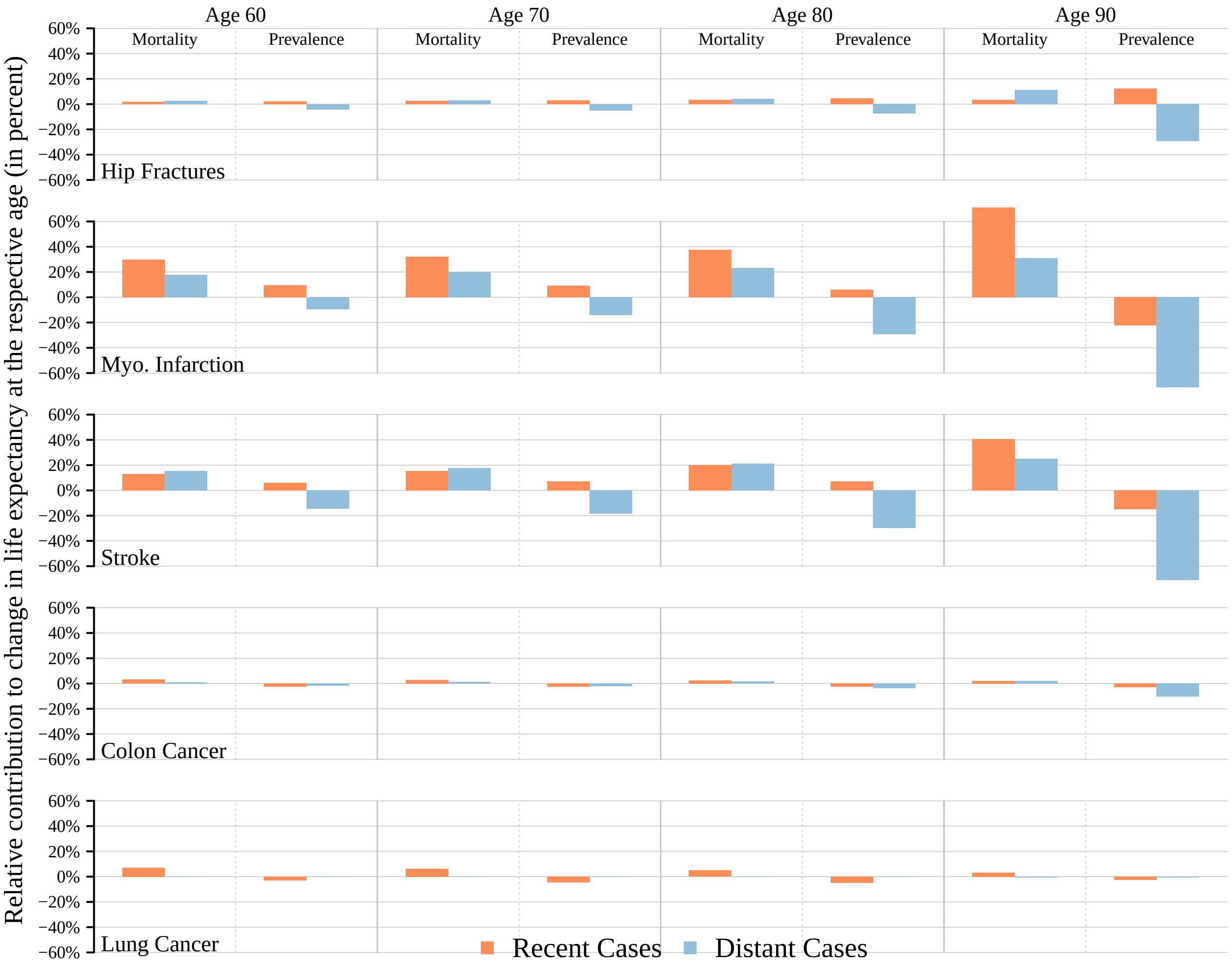

Figure 2: **Decomposition for the difference in remaining life expectancy at ages 60, 70, 80 and 90 between 1994 and 2016 for different diseases, males, Sweden.** The different decompositions rely on different starting ages, and decompose the respective average person-years between the starting age and age 104. For hip fracture, stroke, and myocardial infarction, the recent cases are defined as those that were diagnosed in the three years prior to the calendar year. For the different types of cancer, the recent cases are defined as those that were diagnosed in the five years prior to the calendar year. Accordingly, the distant cases are those in the respective residual group. Each number is the sum across of the age-specific contributions. Data: Swedish National Patient Register and Swedish National Cancer Register. Own calculations.

Mortality dynamics

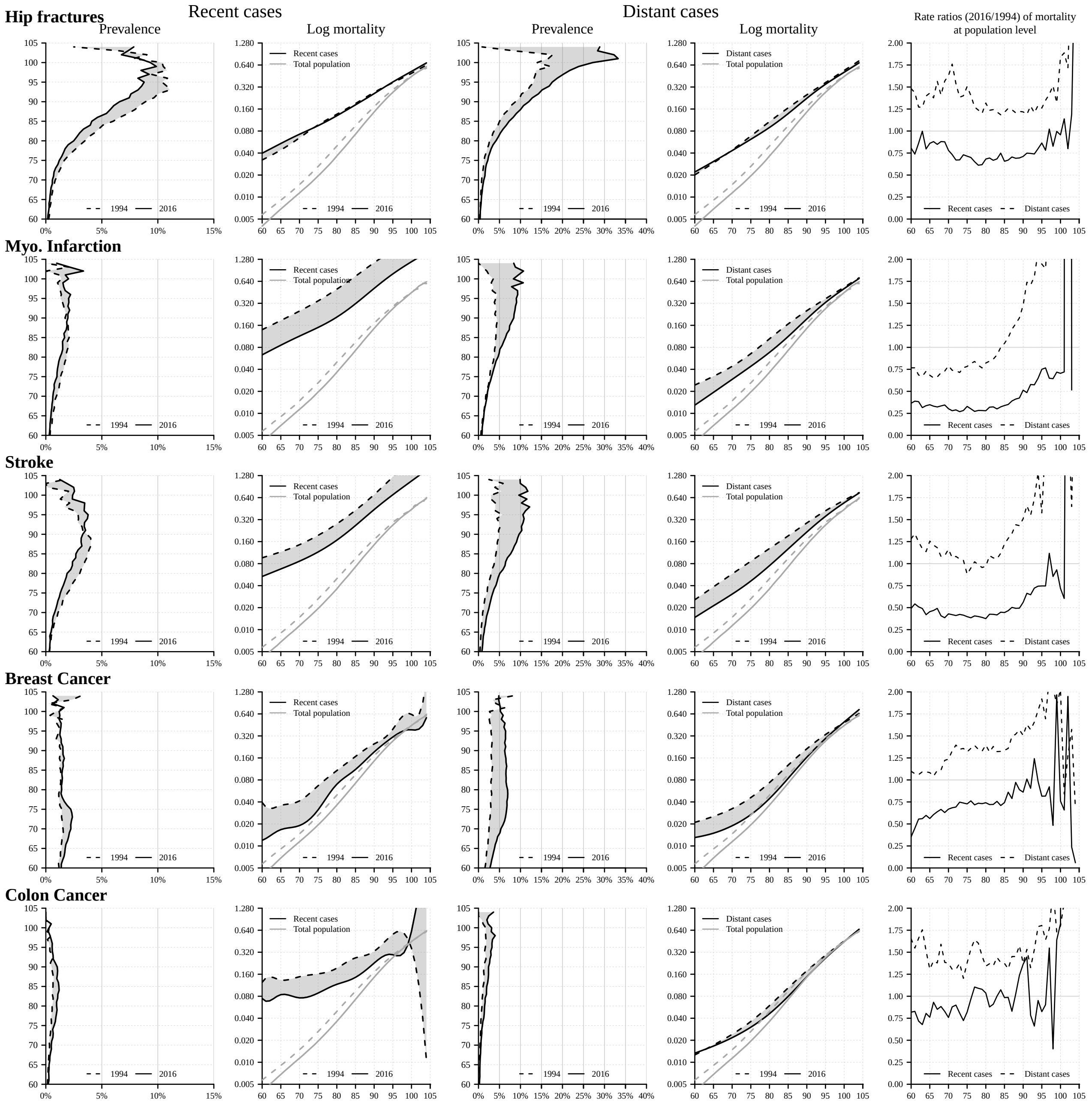

Figure 3: **Development of decomposition components between 1994 and 2016 for different diseases, ages 60–104, females, Sweden.** Death rates have been smoothed with a two-dimensional P-Spline approach.[3] For hip fracture, stroke, and myocardial infarction, the recent cases are defined as those that were diagnosed in the three years prior to the calendar year. For the different types of cancer, the recent cases are defined as those that were diagnosed in the five years prior to the calendar year. Accordingly, the distant cases are those in the respective residual group. Each number is the sum across of the age-specific contributions. Data: Swedish National Patient Register and Swedish National Cancer Register. Own calculations.

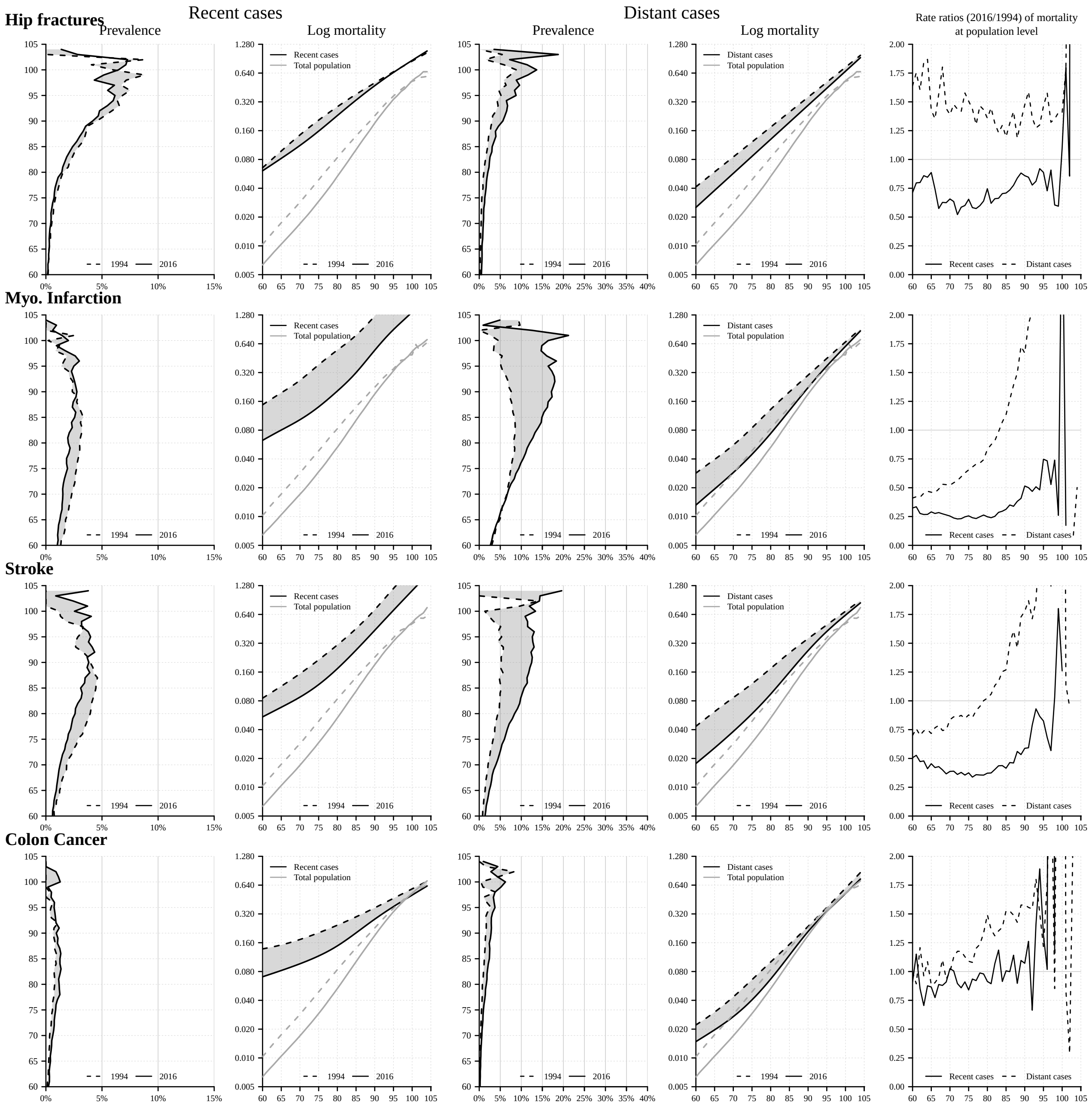

Figure 4: **Development of decomposition components between 1994 and 2016 for different diseases, ages 60–104, males, Sweden.** Death rates have been smoothed with a two-dimensional P-Spline approach.[3] For hip fracture, stroke, and myocardial infarction, the recent cases are defined as those that were diagnosed in the three years prior to the calendar year. For the different types of cancer, the recent cases are defined as those that were diagnosed in the five years prior to the calendar year. Accordingly, the distant cases are those in the respective residual group. Each number is the sum across of the age-specific contributions. Data: Swedish National Patient Register and Swedish National Cancer Register. Own calculations.

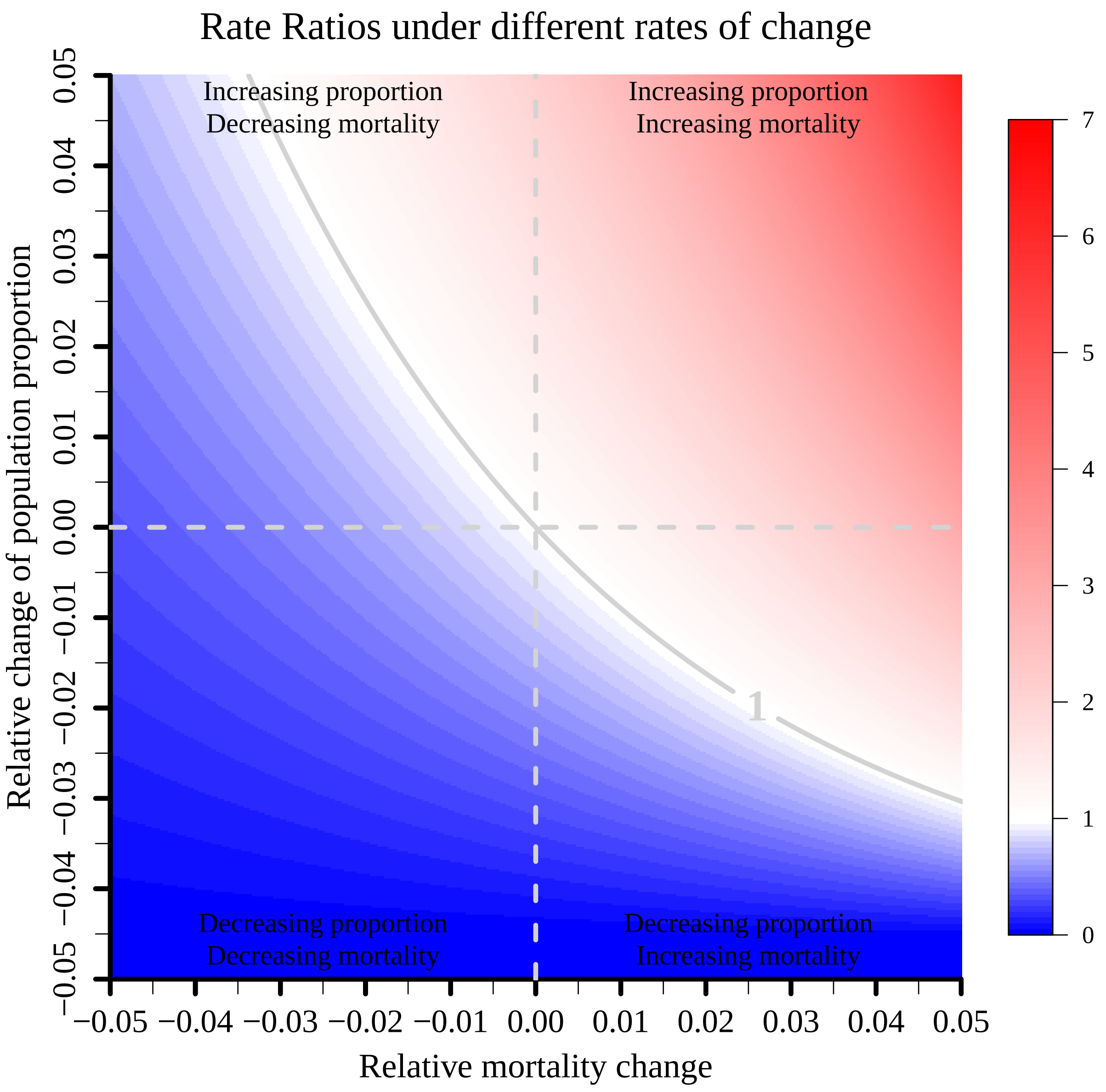

Figure 5: **Simulated rate ratios assuming an exponential change in mortality and a linear change in the population proportion/prevalence with varying rates of change over a time horizon of 23 years.** Levels above one indicate a rise in mortality over time, and vice versa. For the simulation, the initial start levels for the death rates and the population proportion/prevalence are not important since they cancel out in mathematical derivation.

References

[1] Samuel H. Preston, Patrick Heuveline, and Michel Guillot. *Demography – measuring and modelling population processes*. Blackwell Publishers, 2001.

[2] Shiro Horiuchi, John R Wilmoth, and Scott D Pletcher. A decomposition method based on a model of continuous change. *Demography*, 45(4):785–801, 2008.

[3] Carlo G Camarda et al. Mortalitysmooth: An r package for smoothing poisson counts with p-splines. *Journal of Statistical Software*, 50(1):1–24, 2012.
